# Is It Always Unethical to Use a Placebo in a Clinical Trial?

**DOI:** 10.1371/journal.pmed.0020072

**Published:** 2005-03-29

**Authors:** Andreas Stang, Hans-Werner Hense, Karl-Heinz Jöckel, Erick H Turner, Martin R Tramèr

## Abstract

Background to the debate: Placebos are used in trials to conceal whether a treatment is being given or not and hence to control for the psychosomatic effects of offering treatment. Placebo-controlled trials are controversial. Critics of such trials argue that if a proven effective therapy exists, a placebo should not be used. But proponents argue that placebo trials are still crucial to prove the efficacy and safety of many treatments.

## Abstract 2

Placebos are used in trials to conceal whether a treatment is being given or not and hence to control for the psychosomatic effects of offering treatment. Placebo-controlled trials are controversial, and our debate examines the controversies

## Andreas Stang, Hans-Werner Hense, and Karl-Heinz Jöckel's Viewpoint: It Is Unethical When a Beneficial Standard Treatment Exists

A better understanding of the aetiology and pathological mechanisms of diseases often results in new ideas for their treatment. It is then necessary to put these ideas to a formal empirical test in a trial setting. The randomized controlled trial (RCT) is the closest that clinical research can get to the experimental situation. In the RCT, patients are assigned at random to an intervention of putative effectiveness, with the aim of minimizing the potential for bias inherent in nonrandomized clinical research settings. The triumphal advance of RCTs is reflected in their prominent role as one of the pillars of evidence-based medicine.

Initially, when there is uncertainty about the efficacy of a new treatment, clinical researchers are advised to compare the experimental intervention with a placebo. Placebo-controlled trials serve to show that a specific treatment has a beneficial effect on defined clinical endpoints beyond that attributable to mere administration of the intervention by medical professionals. Thus, the early trials of antihypertensive medications and statins were placebo-controlled and were considered to be proof of their beneficial effects.

But what about the next phase? What happens when a treatment for a certain condition, such as hypertension, has been shown to be effective in placebo-controlled RCTs but a newer intervention has been developed for that condition? Let us assume that there is evidence from basic and early clinical trials that the new intervention has a biological effect and has no major side effects in appropriate doses. Should the researchers test it against placebo to prove the superiority of the new treatment?

It is arguably unethical to withhold a therapy of proven efficacy from any patient in a research trial just for the purpose of increasing scientific knowledge. Paragraph 29 of the Declaration of Helsinki states: “The benefits, risks, burdens and effectiveness of a new method should be tested against those of the best current prophylactic, diagnostic, and therapeutic methods” [1]. A note of clarification for paragraph 29 states: “The World Medical Association hereby reaffirms its position that extreme care must be taken in making use of a placebo-controlled trial and that in general this methodology should only be used in the absence of existing proven therapy” [1].

Rothman and Michels have argued that the declaration should include specific examples showing how placebo trials are unethical: “It might suggest as one such example that even in studies of new analgesics to study relief from pain such as headache, the new remedies should be compared only with existing analgesics, and never with placebo. The example will reinforce the point that this principle is not a blurry boundary” [2].

Critics of the declaration argue that forbidding placebo trials puts the manufacturers of a new treatment at a scientific and commercial disadvantage. The manufacturers of a new treatment, say the critics, have to prove that their treatment is as good as an existing one, whereas the manufacturers of the existing treatment had to pass a “lesser test” (superiority over placebo) to get their drug on the market.

For practitioners, though, the crucial question in evaluating a new treatment is how it compares with the standard available treatment, and not whether it is better than placebo. So the important issue is to decide when it is that we can call a therapy “standard”—that is, when can we speak of an indisputable benefit that would make a currently available treatment's use in a trial control group ethically imperative?

Clinical guidelines or recommendations based on high-quality evidence sometimes exist to support use of such a therapy. In situations where no such guidance exists, it is important to assess both the benefits of the therapy (for example, in terms of survival, and relative and absolute risk reduction) and possible harms (including side effects, impaired quality of life, and economic costs). There may be therapies that prolong survival (there is a “gross benefit”) but that cannot be considered to be beneficial because the adverse effects cancel out any survival benefit (there is no “net benefit”). Such therapies cannot be considered “standard” treatment.

One framework for grading the quality of evidence and strength of recommendations on any treatment was published last year by the GRADE working group [3]. The framework stresses the need for judgments based on a formally structured consideration of the balance between benefits and harm, the quality of the evidence, translation of the evidence in specific clinical situations or settings, and the certainty of baseline risks, including resource utilisation.

So when is use of a placebo trial unethical? It is unethical if, in accordance with an assessment similar to the one suggested by the GRADE working group (that is, balancing gross and net benefits in a given trial through a transparent and formalised process), therapies other than the experimental one are judged to be beneficial and are available. In the many situations where such a decision is not clear-cut, the use of placebo may be considered ethically appropriate.

## Erick Turner and Martin Tramèr's Viewpoint: It Can Be Unethical *Not* to Use Placebo

It is generally agreed that placebo is unethical when its use is likely to result in irreversible harm, death, or other serious morbidity. A common argument against placebo is that its use is unnecessary, and therefore unethical, when “proven effective therapy” exists, in which case any new treatment should be tested against this existing treatment. The argument is that if a study drug appears to perform at least as well as a drug that has already been “proven effective”, then the study drug must be effective as well.

The problem with this reasoning is that drug efficacy is not a simple all-or-none matter. If a drug with historical evidence of efficacy could be relied upon to be unfailingly effective—and placebo unfailingly ineffective—in all future clinical trials, we would readily admit that placebo is unnecessary and therefore unethical.

The reality is that “proven effective therapy”—better called “assumed effective therapy” (AET)—often fails to show superiority to placebo. This is not because these drugs are in fact ineffective, but because the trials in question lack assay sensitivity [4,5]. Assay sensitivity is defined as the ability of a trial to distinguish an effective from an ineffective therapy.

Unfortunately, the extent of this problem is poorly appreciated because of publication bias: the tendency for studies that are positive to be published and the tendency of negative and indeterminate studies never to see the light of day [6]. Thus, the myth of infallible “proven therapy” is sustained. But, like a mirage, it vanishes on closer examination.

Khan et al. gained access to unpublished, as well as published, clinical trials data on antidepressants from the Food and Drug Administration (FDA) via the United States Freedom of Information Act [7]. They obtained the FDA review documents on 51 clinical trials on nine antidepressants approved between 1985 and 2000. Of 92 active treatment arms (all involving doses that were eventually approved), 47 (51%) failed to demonstrate statistical superiority to placebo. Of these, there were seven cases (15%) in which the placebo arm was actually superior to AET. Thus, it can be seen that the phrase “proven effective therapy” should be taken with a grain of salt.

Now, what if the FDA had not had the benefit of looking at the placebo arms and relied on an equivalence or noninferiority design [8] comparing study drug with AET? Khan et al. list 12 flexible-dose studies in which (now-approved) study drug outperformed AET (previously approved antidepressants) [7]. Many opponents of placebo would argue that each of these 12 trials provides ample evidence for efficacy of the study drug. However, because these trials did include placebo arms, we discover that in four of them (33%), neither AET nor study drug beat placebo. (In fact, in two of these four trials, AET was numerically inferior to placebo.) Therefore, in these four antidepressant trials, the two “active” drugs were not equally effective, but rather equally ineffective. This critical distinction would have been lost without placebo, and it would have been impossible to ascertain that these seemingly positive trials were in fact false positive trials.

The problem of assay sensitivity is not confined to antidepressants or even to psychotropic drugs in general. A meta-analysis by Tramèr et al found that, among 52 possible comparisons between the “proven” antiemetic ondansetron and placebo, 19 (37%) failed to show a difference [5]. Additionally, many drug classes have shown problems with assay sensitivity (Box 1). The potential for reaching erroneous conclusions by omitting placebo also exists outside of drug studies, as in the example regarding the usefulness of prophylactic respiratory physical therapy on pulmonary function after cardiac surgery [9]; in this specific case, the placebo would be a no intervention control.

**Box 1. Drug Classes That Have Shown Problems with Assay Sensitivity**

- Analgesics
- Antiemetics
- Anxiolytics
- Antihypertensives
- Hypnotics
- Antianginal agents
- Angiotensin-converting enzyme inhibitors for heart failure
- Beta-blockers given after myocardial infarction
- Antihistamines
- Nonsteroidal asthma prophylaxis
- Motility-modifying drugs for gastroesophageal reflux disease

If we were to rely on equivalence or noninferiority designs in studying drugs for indications for which assay sensitivity cannot be assumed, we would risk approving ineffective drugs. It is conceivable that even placebo itself could be approved under such conditions.

According to the Declaration of Helsinki, “Medical research is only justified if there is a reasonable likelihood that the populations in which the research is carried out stand to benefit from the results of the research” [1]. To approve ineffective drugs based on flawed science and to let them loose on an unsuspecting public would be unethical. This is akin to the phenomenon of hypercorrection, in which, in trying very hard to be grammatically correct, the person ends up being grammatically incorrect [10]. In this case, by trying very hard to be ethical and adhering too rigidly to the anti-placebo dogma, one can end up being unethical.

In order to best serve the public health, we must ensure that our clinical drug trials yield scientifically valid results. Where assay sensitivity can be guaranteed, equivalence or noninferiority trials omitting placebo may be ethically preferable. However, where assay sensitivity cannot be guaranteed—and this problem is probably more widespread than we yet realize—difference-showing superiority studies, usually involving placebo, either as monotherapy or add-on therapy, are ethically preferable.

## Stang, Hense, and Jöckel's Response to Turner and Tramèr's Viewpoint

There is some common agreement between Turner and Tramèr's viewpoint and ours—we agree that it is unethical to use placebo when a proven effective therapy exists. However, we question their very narrow definition of “proven effective therapy”. In their definition, a placebo is unethical when the proven effective therapy “could be relied upon to be unfailingly effective—and placebo unfailingly ineffective—in all future clinical trials”. It is possible to argue that all empirical evidence or knowledge is temporary and uncertain. Replication carries no implication for validity [11]. Corroborated hypotheses—in this case, about the effectiveness of a drug—merely “survive” and the degree of corroboration depends on the number and “severity” of tests the hypothesis has survived [12].

We are therefore left with the difficult task of having to evaluate the current evidence of the effectiveness of available treatments in order to decide whether placebo is ethical or not. In other words, we have to make some evaluation on what constitutes “proven effective therapy” based on our current knowledge. The evaluation of current evidence cannot protect us against misinterpretations in the light of future evidence.

It appears to us that Turner and Tramèr think that superiority of a new drug to a control drug could only be established if *all* trials consistently show a statistically significant superiority of the new drug over the control. But studies that evaluate the effectiveness of a new drug may not show identical results for several reasons. Features of the study design, including sample size, dosage, patients' inclusion and exclusion criteria, choice of active control treatment, quality of study conduct, patients' compliance, and other factors can all have an influence on the trial results. Therefore, the proportion of trials showing statistically significant superiority (bullet counting) is an inappropriate indicator of drug superiority and a proportion less than 100% is no indicator of lack of superiority. Several analytical techniques, including meta-analysis and meta-regression, that account for design features are available and provide better insights into the superiority of drugs than bullet counting.

## Turner and Tramèr's Response to Stang, Hense, and Jöckel's Viewpoint

Stang and colleagues quote from part of a clarification to the Declaration of Helsinki. But the clarification continues: “a placebo-controlled trial may be ethically acceptable, even if proven therapy is available…where for compelling and scientifically sound methodological reasons its use is necessary to determine the efficacy or safety of a prophylactic, diagnostic or therapeutic method” [1]. Our viewpoint was essentially an evidence-based discussion of this clarification and its ethical implications. Assuming that medical research successfully rids itself of publication bias [13,14], it should become increasingly obvious that, for many drug classes (Box 1), the emperor of “proven therapy” is wearing no clothes [15].

But this debate is not only about efficacy; it is also about harm. In the absence of a placebo group, it may be impossible to interpret a drug's potential for harm. Let us look at Stang and colleagues' example of analgesics. The Vioxx Gastrointestinal Outcomes Research (VIGOR) trial showed a five-fold difference in the incidence of myocardial infarction in the rofecoxib (Vioxx) group compared with the naproxen group [16].

Nonsteroidal anti-inflammatory drugs such as naproxen, however, inhibit platelet function and therefore might have a myocardial protective effect [17]. Since the VIGOR trial did not include a placebo group, it remained unclear whether there was an increased risk of myocardial infarction with rofecoxib or a decreased risk with naproxen. Four years later, and after tens of millions of patients had received rofecoxib [18], Merck announced they were withdrawing the drug because of an increased cardiovascular risk [19]. The decision was based on the unpublished Adenomatous Polyp Prevention on Vioxx (APPROVe) study, a placebo-controlled three-year trial of rofecoxib.

In his November 2004 testimony before the United States Senate, David Graham of the FDA provided an estimate of the rate of excess cases of Vioxx-related myocardial infarction and sudden cardiac death. He testified that it was as if, for the five years that Vioxx was on the United States market, “2 to 4 jumbo jetliners were dropping from the sky every week” [20]. Of those cases, he added, 30% to 40% probably died.

If those who believe that “proven therapy” trials are ethically preferable to placebo-controlled trials had had their way, the APPROVe study would have been blocked, and Vioxx would still be on the market today. It seems ironic that such a stance could be taken in the name of ethics.

**Figure:**
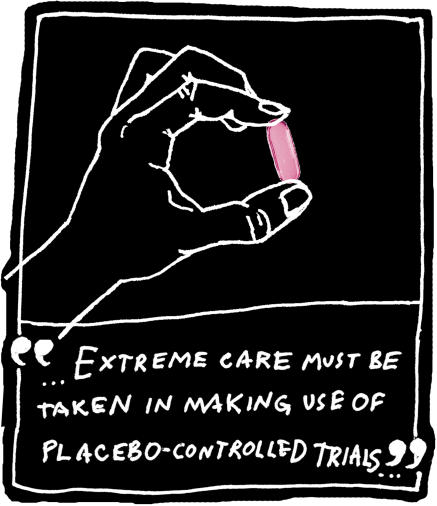
(Illustration: Margaret Shear, Public Library of Science)

**Figure:**
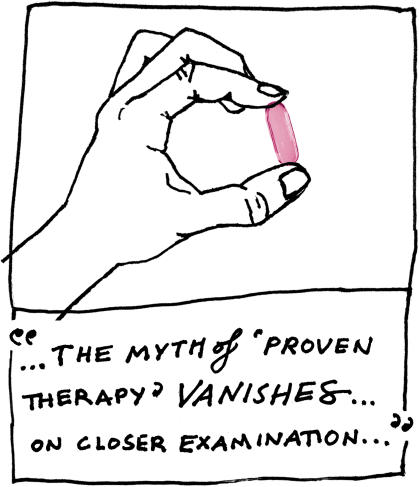
(Illustration: Margaret Shear, Public Library of Science)

## Abbreviations

AET: assumed effective therapy
APPROVe: Adenomatous Polyp Prevention on Vioxx
FDA: Food and Drug Administration
RCT: randomized controlled trial
VIGOR: Vioxx Gastrointestinal Outcomes Research
